# Integrating *In Silico* and *In Vitro* Analysis of Peptide Binding Affinity to HLA-Cw*0102: A Bioinformatic Approach to the Prediction of New Epitopes

**DOI:** 10.1371/journal.pone.0008095

**Published:** 2009-11-30

**Authors:** Valerie A. Walshe, Channa K. Hattotuwagama, Irini A. Doytchinova, MaiLee Wong, Isabel K. Macdonald, Arend Mulder, Frans H. J. Claas, Pierre Pellegrino, Jo Turner, Ian Williams, Emma L. Turnbull, Persephone Borrow, Darren R. Flower

**Affiliations:** 1 The Jenner Institute, University of Oxford, Compton, Berkshire, United Kingdom; 2 Department of Immunohaematology and Blood Transfusion, Leiden University Medical Centre, Leiden, The Netherlands; 3 Centre for Sexual Health and HIV Research, Royal Free and University College London Medical School and Camden Primary Care Trust, London, United Kingdom; University of Toronto, Canada

## Abstract

**Background:**

Predictive models of peptide-Major Histocompatibility Complex (MHC) binding affinity are important components of modern computational immunovaccinology. Here, we describe the development and deployment of a reliable peptide-binding prediction method for a previously poorly-characterized human MHC class I allele, HLA-Cw*0102.

**Methodology/Findings:**

Using an in-house, flow cytometry-based MHC stabilization assay we generated novel peptide binding data, from which we derived a precise two-dimensional quantitative structure-activity relationship (2D-QSAR) binding model. This allowed us to explore the peptide specificity of HLA-Cw*0102 molecule in detail. We used this model to design peptides optimized for HLA-Cw*0102-binding. Experimental analysis showed these peptides to have high binding affinities for the HLA-Cw*0102 molecule. As a functional validation of our approach, we also predicted HLA-Cw*0102-binding peptides within the HIV-1 genome, identifying a set of potent binding peptides. The most affine of these binding peptides was subsequently determined to be an epitope recognized in a subset of HLA-Cw*0102-positive individuals chronically infected with HIV-1.

**Conclusions/Significance:**

A functionally-validated *in silico-in vitro* approach to the reliable and efficient prediction of peptide binding to a previously uncharacterized human MHC allele HLA-Cw*0102 was developed. This technique is generally applicable to all T cell epitope identification problems in immunology and vaccinology.

## Introduction

The products of the Major Histocompatibility Complex (MHC) play a fundamental role in regulating immune responses, modulating the functional development of lymphocyte subsets, the acquisition and maintenance of self-tolerance, and the activation state and responses of host immune defences. MHC class I molecules expressed on the cell surface report on the internal status of cells by presenting ligands for surveillance by CD8+ T cells, natural killer T (NKT) cells and natural Killer (NK) cells [**1**]. CD8+ T cells recognise antigen as short peptide fragments complexed with classical MHC class I molecules [**2**]. NK cells express a diverse array of receptors that interact with ligands including classical and non-classical MHC class I molecules, which exert positive and negative influences on their functions [**3**]. Human MHC class I molecules are both polygenic and highly polymorphic [**4**]. This increases the chance that every pathogen will contain many epitopes recognised by individuals within the population and places restraints on a pathogen's ability to escape immune control.

Characterisation of the peptides that are presented by MHC molecules is of tremendous utility in basic research studies, and can also have clinical applications. Identification of the ligands recognised by T cells and NK cells facilitates analysis and manipulation of lymphocyte subsets participating in host defence and in disease processes, and can help mediate the development of immune-based prophylactic and therapeutic strategies including vaccines. Immunoinformatics, a newly emergent sub-discipline of bioinformatics, addresses informatic problems within immunology, such as the crucial issue of epitope prediction [**5**]. As high throughput biology reveals the genomic and proteomic sequences of pathogenic bacteria, viruses, and parasites, such prediction will become increasingly important in the post-genomic discovery of novel vaccines, clinical diagnostics, and laboratory reagents. Direct laboratory-based analyses of T cell responses to overlapping peptides drawn from pathogen proteomes are expensive in terms of time, labour, and resource. The accurate prediction of peptide-MHC binding provides a useful approach to candidate T cell epitope selection since it allows the number of experiments needed for their identification to be minimised. Database-driven models of peptide binding include multivariate methods such as partial least squares (PLS) and artificial neural networks [**6**] **,** [**7**] **,** [**8**] **,** [**9**].

To better understand the sequence-dependence of peptide-MHC binding, we have taken a novel approach to exploring the amino acid preferences of various human and mouse MHC alleles [**10**]. Our approach to determining epitope-mediated immunogenicity encompasses an integrated system comprising a state-of-the-art database system known as AntiJen [**11]**, [12], [13****] and the quantitative structure-activity relationship (QSAR)-based prediction of binding to class I [**14**] and class II molecules [**15**], coupled to integrated experimental validation [**10**]. We have deployed our QSAR prediction models via MHCPred [**16**]; subsequently supplementing this with sophisticated models of antigen presentation [**17**]; deployed via EpiJen [**18**].

At the heart of our work is an immunoinformatic technique for the prediction of peptide-MHC affinities, commonly known as the additive method [**19**]. It is a two-dimensional quantitative structure-activity relationship (2D-QSAR) technique whereby the presence or absence of a group is correlated with biological activity. For a peptide, the binding affinity is thus represented as the sum of amino acid contributions at each position. Notably, using cell surface MHC stabilisation assays to experimentally determine peptide MHC binding affinities, we have used the additive method to drive validation of our predictions and the manipulation of peptide specificity for MHC alleles, leading to the discovery of HLA-A*0201 superbinding peptides and potential HLA-A*0201-presented epitopes which lack canonical anchors [**10**].

Here we use similar methodology to characterise the peptide binding specificity of the human MHC class I allele HLA-Cw*0102. Study of the HLA-C alleles and the peptides they present has received much less attention than work on HLA-A and -B alleles. This is likely due to the fact that they are expressed at lower levels on the cell surface than HLA-A and -B alleles [**20**], and a higher proportion of CD8+ T cell responses are believed to be restricted by HLA-B and HLA-A, with HLA-C a poor third [**21]**, [22****]. However despite this, HLA-C-restricted CD8+ T cell responses can still constitute immunodominant components of the host T cell response, and can exert significant immune pressure on *in vivo* pathogen replication [**23]**, [24****]. Further, in addition to interacting with antigen-specific receptors on CD8+ T cells, HLA-C molecules also interact with other activating/inhibitory receptors that regulate the maturation and functions of lymphocyte subsets including NK cells, being more important than HLA-A alleles and certain HLA-B alleles in this regard [**3]**, [25****]. The HLA-C1 group of alleles (to which HLA-Cw*0102 belongs) interact with killer cell immunoglobulin-like receptors (KIRs), specifically inhibitory receptors KIR2DL2 and KIR2DL3 and activating receptor KIR2DS2, while the HLA-C2 group of alleles interact with the inhibitory receptor KIR2DL1 and the activating receptor KIR2DS1 [**26**]. The importance of HLA-C in the host immune response, which was previously underestimated, is thus now increasingly appreciated. This was emphasised recently by results from a genome-wide association study of major determinants of host control of HIV infection, which identified a polymorphism located near to the HLA-C gene that is thought to associate with differences in HLA-C expression levels as an important determinant of set-point plasma viremia [**27**].

Unlike many HLA-C alleles, peptide binding to HLA-Cw*0102 has been investigated previously [**23]**, [28****], but not with any great rigour or precision. Barber *et al*. [**29**] combined results from pooled sequencing of eluted cell-surface peptides and the sequencing of individual peptides using Edman degradation, as confirmed with tandem mass spectrometry, to propose a motif for HLA-Cw*0102. Their reading of the data suggested strong amino acid preferences at six of the nine peptide positions of the nonameric peptide. Guided perhaps by a implicit structural understanding and precedents established by other alleles, the motif presented at SYFPEITHI [**30**] is much simplified: Ala and Leu at position P2 and Leu at the C-terminus (P9) of the epitope peptide.

Sequence motifs are the oldest and currently still the best-known tools for predicting the peptide-specificity of allele-dependent MHC-peptide binding. Motifs are characterized by a few dominant anchor positions with a very restricted set of allowed amino acids. Such anchors are thought essential for binding. The best-understood human MHC class I allele is HLA-A*0201 [**10**]. For a nonameric peptide, it has anchor residues at peptide positions P2 (accepting Leu and Met) and P9 (accepting Val and Leu). The motif method is admirably simple: it is easy to implement either by eye or more systematically by using a computer to scan through protein sequences. However simple motif-based approaches are unable to provide complete or very accurate predictions of the peptides able to bind to a particular MHC class I molecule. Andersen *et al*. [**28**] used a semi-quantitative cell-surface stabilisation binding assay for HLA-Cw*0102 to measure the affinity of 20 peptides initially identified using a motif modified from that of Barber *et al*. [**29**]. Of the 20 motif- positive peptides, only 12 exhibited measurable affinity. Notably, the few HIV-1-derived CD8+ T cell epitopes restricted by HLA-Cw*0102 that have been identified to date were determined by screening of overlapping peptides. Using this approach, Goulder et al [**23**] identified the octameric HIV-1 Gag peptide VIPMFSAL as a T cell epitope. More recently, Liu *et al*. [**31**] were able to identify several more HLA-Cw*0102-restricted HIV epitopes: NSPTRREL, YSPLSLQTL, YCAPAGFAIL, and HAPWDVNDL.

Due to the paucity of experimental data, peptide-binding prediction methods for HLA-C including HLA-Cw*0102 have received little attention, and, until recently, were not generally available. As methods are developed that aim at extending prediction beyond the small number of well-characterised MHC class I alleles, several computational approaches have begun to explore HLA-C in general and HLA-Cw*0102 in particular [**32]**, [33], [34], [35****]. In this study, rather than take a solely theoretical approach, we sought to combine computational and experimental methodologies, developing a hybrid *in silico-in vitro* algorithm for peptide-HLA-Cw*0102 binding prediction. Using an in-house, fluorescence-activated cell sorting (FACS) -based MHC stabilization assay [**36**], we determined the binding affinities to the MHC class I allele HLA-Cw*0102 of a set of 43 nonameric peptides. An additive QSAR model was developed from this data, which we used to reassess the preferred amino acids at each position and to design new HLA-Cw*0102-binding peptides. Moreover, in addition to undertaking *in vitro* tests, we used our model to try to predict HIV-1 T cell epitopes restricted by HLA-Cw*0102. Reactivity to the most affine of the candidate peptides (CAPAGFAIL) was subsequently observed in HIV-infected individuals, confirming its status as a HIV-1 epitope.

## Results

### Development of an Additive 2D-QSAR Model for Binding of Nonameric Peptides to HLA-Cw*0102

To generate data for use in developing a computational model of peptide binding to HLA-Cw*0102, 43 peptides identified from the work of Barber *et al*. [**28**] and Anderson *et al.* [**23**] (Table 1) were assayed for binding to HLA-Cw*0102 using a FACS-based cell surface MHC stabilisation assay [**10]**, [19****]. This identified 25 peptides that bound to HLA-Cw*0102 with different affinities (peptides 1–25, Table 1). The experimentally-determined pBL_50_ values ranged from over 7 to 3; peptides with pBL_50_ values below 3 were considered to be non-binders. From this set of binding peptides, two models were generated using the PLS-based additive method [**19**]: one containing just amino acid contributions and one with contributions from both amino acids and side chain–side chain interactions. The two additive models were roughly equivalent in terms of statistical quality, so we applied the principle of Occam's razor and sought the simplest explanation, choosing the amino acid only model. The amino acid only model was tested using cross-validation: predicted peptide binding affinities are shown in Table 1. The non-cross-validated parameters were *r* ^2^ = 0.920 and standard error of estimate (SEE)  = 0.299. Leave-one-out cross-validation (LOO-CV) gave *q* ^2^ = 0.491, standard error of prediction (SEP)  = 0.753, number of components (NC)  = 2, and no outliers were removed. These data are consistent with a robust model. Notably, there was a trend for the experimentally-measured peptide binding affinities to be slightly higher than the values predicted by the model; potential reasons for this are discussed below.

**Table 1:** List corresponding to the initial set of peptides used in this study and their experimentally-determined and predicted HLA-Cw*0102 binding affinities.

No.	PEPTIDE	Ref.	pBL_50(exp)_ a	pBL_50(pred)_ b
1	A A P A Y S R A L	21	5.751	6.010
2	F A P Y N K P S L	21	6.214	6.570
3	I T P T G T H P L	21	6.548	6.290
4	L P E T K F S E L	21	5.196	5.280
5	N A P W A V T S L	21	6.848	6.880
6	N C P E R I I T L	21	7.198	7.120
7	V A P W N S L S L	21	6.153	6.380
8	A Q P Q T T T P L	22	6.915	6.690
9	A T S P I V P S L	22	5.382	5.620
10	E V I P M F S A L	22	6.131	5.780
11	F A P G N Y P A L	22	6.723	6.510
12	I L R R P T S P V	22	5.099	5.110
13	I L S P L T K G I	22	3.061	3.410
14	I L S P R K E S V	22	4.764	4.680
15	I L S P R S E S V	22	5.581	5.050
16	I L S P R S K E S	22	3.534	4.460
17	I L S P S K E S V	22	4.944	4.390
18	L L T S P D V G L	22	4.933	5.140
19	L S P L T K G I L	22	5.421	5.050
20	L S P R S K E S V	22	6.041	6.330
21	L T S P D V G L L	22	4.563	5.180
22	P L S P P K K K D	22	4.996	5.350
23	S L E E C D S E L	22	3.426	3.770
24	V I P M F S A L S	22	5.610	5.440
25	Y A Q P Q T T T P	22	5.225	5.380
26	H L P E T K F S E	21	Non-binder^C^	
27	A D A E K P F Y V	22	Non-binder^C^	
28	A D D S H F V S I	22	Non-binder^C^	
29	A V D A D D S H F	22	Non-binder^C^	
30	D A D D S H F V S	22	Non-binder^C^	
31	D L L T S P D V G	22	Non-binder^C^	
32	G A D A E K P F Y	22	Non-binder^C^	
33	G I P E P A H A Y	22	Non-binder^C^	
34	I L K E P V H G V	22	Non-binder^C^	
35	I L S R S K E S V	22	Non-binder^C^	
36	I P E P A H A Y A	22	Non-binder^C^	
37	K A T S P I V P S	22	Non-binder^C^	
38	L L K L A S P E L	22	Non-binder^C^	
39	L P S Q A M D D L	22	Non-binder^C^	
40	L S P P K K K D L	22	Non-binder^C^	
41	P L P S Q A M D D	22	Non-binder^C^	
42	V D A D D S H F V	22	Non-binder^C^	
43	Y M T P S S R P L	22	Non-binder^C^	

a Experimental pBL_50_s measured using a FACS-based MHC stabilisation assay.

b Calculated pBL_50_s generated using the QSAR model described in the text.

C Experimental non-binders were initially predicted to be non- or low binding peptides, but without useful discriminatory power.

**Figure 1:**
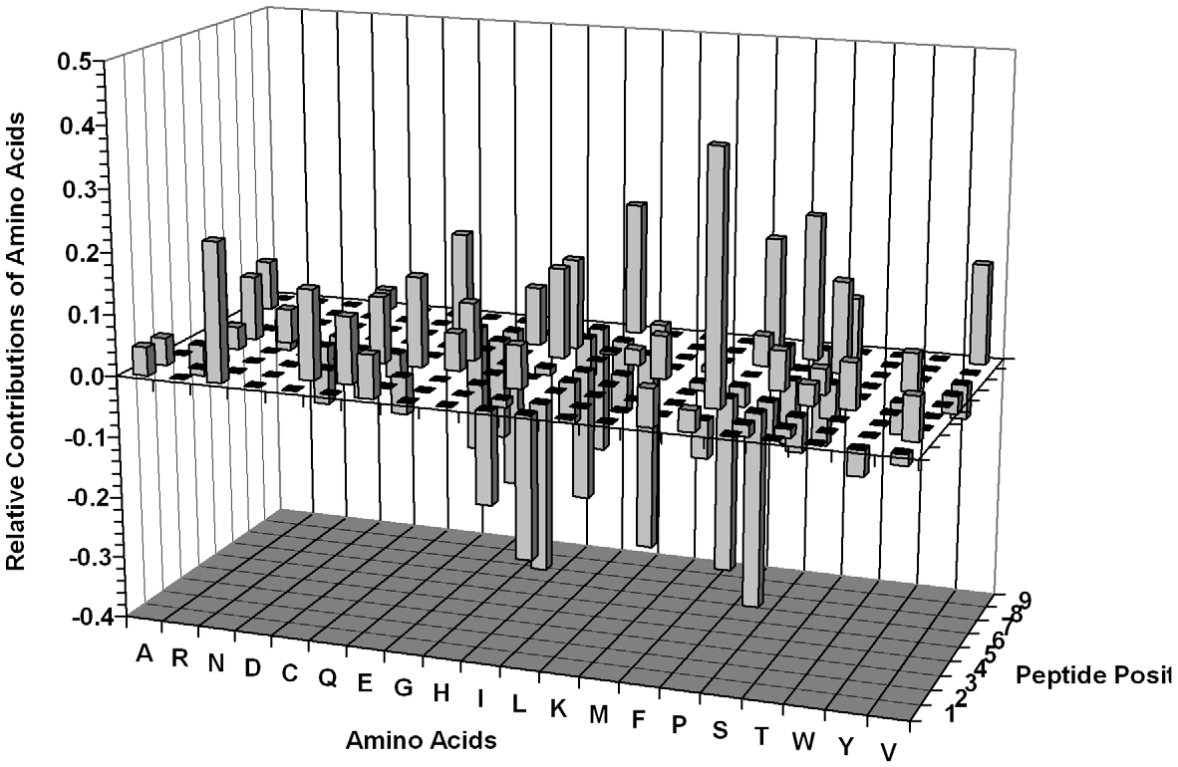
Relative contributions of different amino acids at each position to the interaction of a nonameric peptide with the HLA-Cw*0201 molecule as determined using a QSAR-based additive model. The bar heights indicate the strength of the positive or negative contribution made by each amino acid residue (denoted using the single letter code) at the indicated position (1–9) in a nonameric peptide to peptide-MHC interaction. The contribution is equivalent to a position-wise amino acid regression coefficient obtained by PLS regression (as described in the text).

The contributions made by different individual amino acids at each position of a generalised nonameric HLA-Cw*0102-binding peptide are presented in Figure 1. Favoured and disfavoured binding residues, as defined by the model (applying a threshold of >±0.1), are shown in Table 2. The amino acids present at each position in the nonameric peptide were found to impact in a positive or negative fashion on peptide binding to HLA-Cw*0102. The positions with the most pronounced effects on peptide-MHC binding clustered at the N- and C-termini of the peptide sequence, consistent with the observation that peptides bound to most MHC class I molecules are typically anchored at or near their termini [**37**].

**Table 2:** Summary of amino acids at each position that favour or disfavour peptide binding to HLA-Cw*0102, as defined by the QSAR model.

	P1	P2	P3	P4	P5	P6	P7	P8	P9
**Favoured Binding** a	**N** b	C, Q	**P**	E, Q	R	I	**E**, I, T	**P**, **S**, T	**L**, V
**Disfavoured Binding** a	I, **L**	**L**	**S**	P	L	S	G, K	E, G, **L**	I, S

a Amino acids are included if they exceed a threshold of >±0.10 as favoured or disfavoured residues as shown in Fig. 1.

b Amino acids are highlighted in bold if they exceed a threshold of >±0.20 as favoured or disfavoured residues as shown in Fig. 1.

### Design of Peptides Optimised for Binding to HLA-Cw*0102

The model described above enumerates the effects of amino acid residues at all positions of a nonameric peptide on peptide binding to HLA-Cw*0102 (Table 2). It was used to design a set of peptides optimised for HLA-Cw*0102 binding, and their MHC binding affinities were then determined experimentally. The peptides designed for optimal binding were prepared by combining preferred amino acids at each position. For certain positions (1, 3, 5, 6) there were single preferred residues, whilst other positions (2, 4, 7, 8, 9) had several acceptable amino acids. We selected favoured amino acid residues from Table 2, combining all preferred residues to generate new peptides. We chose Asn for position 1 (P1); Cys and Gln for P2; Pro for P3; Glu and Gln for P4; Arg for P5; Ile for P6; Glu, Ile and Thr for P7; Pro, Ser and Thr for P8; and Leu and Val for P9. Combining preferred amino acids produced 256 peptides. Peptide binding affinities were predicted, and the affinities of 4 distinct high binders were determined using flow-based MHC stabilization assays (Table 3a). A good correlation was found between the experimentally-determined and predicted affinities, r^2^_(pred)_ = 0.919. These peptides all bound strongly to HLA-Cw*0102. However the binding affinities of these peptides did not exceed substantially that of NCPERIITL, the most affine peptide tested in the original peptide set (pBL_50(exp)_ = 7.120). Additional experiments were thus conducted to gain further insight into the impact of amino acids at different positions in the sequence on peptide binding to HLA-Cw*0102.

**Table 3:** Test sets of newly-designed peptides.

No.	Peptide	pBL_50__(pred)_ a	pBL_50__(exp)_ b
**(a) Optimized binders**			
**1**	NCPEGYTSL	7.159	7.140
**2**	NEPQRIEPL	7.051	7.017
**3**	EQPERYESV	6.947	6.879
**4**	ECPQGITPV	6.954	6.962
**(b) Alanine Substitutions**			
	NCPERIITL		**7.120**
**5**	ACPERIITL	6.941	6.734
**6**	NAPERIITL	7.020	8.503
**7**	NCAERIITL	6.717	6.363
**8**	NCPARIITL	6.924	6.943
**9**	NCPEAIITL	7.097	6.714
**10**	NCPERAITL	6.975	6.613
**11**	NCPERIATL	6.963	6.696
**12**	NCPERIIAL	7.097	6.852
**13**	NCPERIITA	6.907	6.590
**(c) Exploring Amino Acid Contributions**			
**14**	YMPTASCDL	-	7.89
**15**	WAPHTDTSL	-	7.90
**16**	VAPQLTFGL	-	7.79
**17**	LGPEKLQYL	-	6.82
**18**	MQPSRGKTL	-	6.77
**19**	HMPDVGCIL	-	6.75
**20**	SWPMQFDAL	-	6.57
**21**	CMPDWLDRL	-	6.48
**22**	TDPWFHRSL	-	6.47
**23**	EFPWVFIEL	-	6.45
**24**	CAPREPHVV	-	6.42
**25**	EAPMWDHWL	-	6.31
**26**	HNPGIATPV	-	6.18
**27**	FMPIMKNEV	-	6.17
**28**	VIPMFSAL	-	6.07
**29**	KSPLDIVNL	-	5.72
**30**	WRPDVNMQL	-	5.54
**31**	WGPGIIWAL	-	5.44
**32**	VRPMRQWPL	-	5.42
**33**	QYPKGDAWV	-	Non-binder

a Calculated pBL_50_s generated using the QSAR model described in the text.

b Experimental pBL_50_s measured using a FACS-based MHC stabilisation assay.

### Use of Alanine Scanning to Explore Systematically the Roles of Amino Acids at Different Positions in Peptide Binding to HLA-Cw*0102

To explore systematically the relative contribution made by amino acids at different positions in a nonameric peptide to HLA-Cw*0102 binding affinity, a sequential alanine-scan of the peptide NCPERIITL, which bound with high affinity to HLA-Cw*0102 (pBL_50_ = 7.12), was undertaken, whereby each of residues 1 to 9 was changed to alanine. The alanine-substituted peptides (Table 3b) were synthesized and their binding to HLA-Cw*0102 was tested in flow-based MHC stabilisation assays. Interestingly, although replacement of key MHC-interacting residues in a peptide with alanine residues typically results in a non-trivial decrease in the affinity of peptide binding to MHC, the predicted HLA-Cw*0102-binding affinities of the alanine-substituted versions of the NCPERIITL peptide were not appreciably different from that of the index peptide (Table 3b). Further, the experimentally-determined binding affinities of these peptides were also not substantially lower than that of the index peptide (Table 3b). Only the conversion of Cys at position 2 produced a marked change in peptide binding affinity: but the effect observed was an increase in peptide binding affinity of 1.2 log units. These results are consistent with the idea that the affinity of peptide binding to HLA-Cw*0102 is determined by interactions made by amino acids at all positions in the peptide sequence, rather than being solely dependent on a single prominent anchor residue.

### Exploration of the Contributions of a Diverse Range of Amino Acids at Each Position to Peptide Binding to HLA-Cw*0102

A potential shortcoming of our approach was that not every amino acid was represented at each of the 9 possible positions in the set of peptides used to generate the model – hence the positive or negative influences of certain amino acids at particular positions may have been overlooked. To gain insight into the extent to which this may have affected the utility of the model, a set of 20 sequence-diverse peptides exploring different amino acids at positions 1, 2, 4, 5, 6, 7, and 8 within the peptide were designed (Table 3c). We chose positions 3 and 9 as pseudo-anchors (on the basis of data in Table 2), and varied amino acids at all other positions. A simple genetic algorithm [**38**] was implemented to create peptide sequences. The genetic algorithm was constrained using several criteria, such as the number of acidic and basic residues in the peptide. Using the protocol outlined in [**39**], the resulting large set of possible peptides was filtered using a discriminant function model that aimed to partition binders from non-binders. This model was built from the initial set of binders and non-binders (as defined by their experimentally measured affinities; see Table 1). The model generated scores for each peptide which represented the probability that they would bind rather than their affinity; thus a weak binder would score as well as a strong binder. Missing values were set to be neutral and peptides were ranked using their resulting binding score. For the top 250 peptides, diversity selection [**40**] was used to choose 20 sequence-diverse peptides for testing (Table 3c). One peptide, with a pBL_50_<3.00, was a non-binder; the rest exhibited appreciable binding to HLA-Cw*0102 (Table 3c). We constructed a revised affinity model including the new peptides. The non-cross-validated parameters were *r* ^2^ = 0.890 and SEE  = 0.271. LOO-CV gave *q* ^2^ = 0.421, SEP  = 0.724, NC = 3. While these results were encouraging, the new model showed no marked improvement over the original model. Nonetheless, our approach [**39**] can draw strong validation from these findings: a very high proportion of peptides were found to bind, and the use of multiple cycles of this strategy should allow large numbers of affine peptides to be discovered efficiently.

### In Silico Discovery of Novel HIV Epitopes

As a test of the utility of our model for prediction of HLA-Cw*0102-binding peptides in a pathogen sequence that may constitute T cell epitopes, we sought to identify HLA-Cw*0102-binding peptides in the sequence of HIV-1 and to determine whether any of these were recognised by the virus-specific T cell response in HLA-Cw*0102-positive individuals infected with this virus. Two full-length HIV-1 proteome sequences were analysed for peptides with predicted high binding affinity for HLA-Cw*0102: the 2001 Clade B consensus sequence (a virtual sequence representing the most frequently observed amino acids at each position in multiple sequence alignments of HIV isolates) and the sequence of the clade B virus HXB2 [**41**]. Twenty-two unique peptides were predicted to possess predicted pBL_50_ HLA-Cw*0102-binding affinities in excess of 5.0, which was deemed sufficient for them to be potential T cell epitopes [**10**]; see Table 4. These twenty-two peptides were evaluated using the flow-based HLA-Cw*0102 binding assay, and half were found to demonstrate measurable HLA-Cw*0102 binding (Table 4). We discuss a number of possible reasons for the lack of demonstrable binding of the other peptides below.

**Table 4:** HIV-1 peptides predicted to bind to HLA-Cw*0102.

Peptide number a	Peptide sequence	HIV Position	pBL_50__(pred)_ b	pBL_50__(exp)_ c
	**Clade B consensus**			
Peptide 4	EVIPMFSAL	Gag 167–175	5.728	6.13116
Peptide 5	VIPMFSALS	Gag 168–176	5.44	5.61006
	IVRMYSPTS	Gag 273–281	5.211	Non-binder
	FPISPIETV	Pol 155–163	6.05	Non-binder
Peptide 6	LTEEKIKAL	Pol 181–189	5.376	5.28447
Peptide 7	FQSSMTKIL	Pol 315–323	5.117	6.15401
Peptide 8	LLRWGFTTP	Pol 364–372	5.363	5.68929
	IVIWGKTPK	Pol 535–543	5.004	Non-binder
	LVSAGIRKV	Pol 706–714	5.344	Non-binder
	PQSQGVVES	Pol 860–868	5.058	Non-binder
	VVPRRKAKI	Pol 974–982	5.915	Non-binder
	LITPKKIKP	Vif 153–161	5.915	Non-binder
Peptide 10	LQILAIVAL	Vpu 4–12	5.826	6.11562
Peptide 9	PVPLQLPPL	Rev 70–78	5.335	5.50055
Peptide 1	CAPAGFAIL	Gp160 218–226	5.116	8.00554
	EQFGNKTIV	Gp160 350–359	5.044	Non-binder
Peptide 2	RVRQGYSPL	Gp160 703–711	5.208	7.06262
Peptide 3	IVTRIVELL	Gp160 773–773	5.617	5.94945
	**HXB2 concensus** d			
Peptide 11	ELQAIYLAL	Pol 633–641	5.529	5.90231
	ELIRTVRLI	Rev 11–19	5.031	Non-binder
	NSTWSTEGS	Gp160 397–405	5.46	Non-binder
	NYTSLIHSL	Gp160 637–645	5.077	Non-binder

a Peptide number used in subsequent ELISPOT experiments.

b Calculated pBL_50_s generated using the QSAR model described in the text.

c Experimental pBL_50_s measured using a FACS-based MHC stabilisation assay.

d Peptides listed here were unique to the HXB2 sequence.

**Figure 2:**
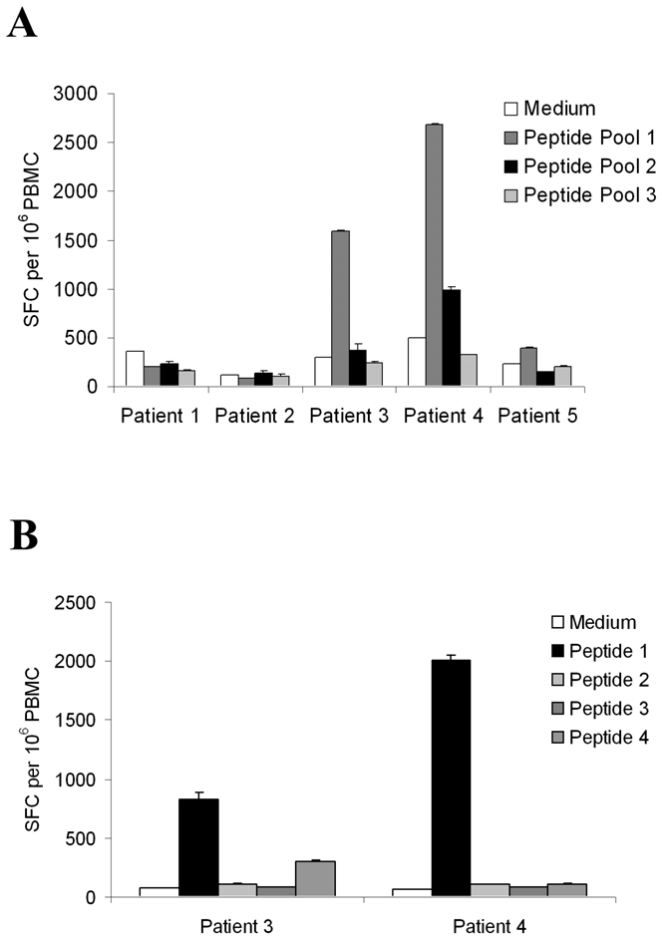
Analysis of the recognition of HLA-Cw*0102-binding HIV-1 peptides by T cells from HLA-Cw*0102-positive HIV-infected individuals. PBMCs from clade B HIV-infected individuals were screened for responses to (a) pools of HLA-Cw*0102-binding peptides (pool 1 =  peptides 1–4; pool 2 =  peptides 5–8; pool 3 =  peptides 9–11) or (b) individual peptides (1–4) by IFN-γ ELISPOT assay. The number of cells producing IFN-γ in response to stimulation with peptide(s) or medium alone is shown, expressed as the mean (of results obtained in duplicate test wells) number of spot-forming cells (SFC) per 10^6^ PBMCs. The error bars indicate 1 standard deviation above the mean.

**Figure 3:**
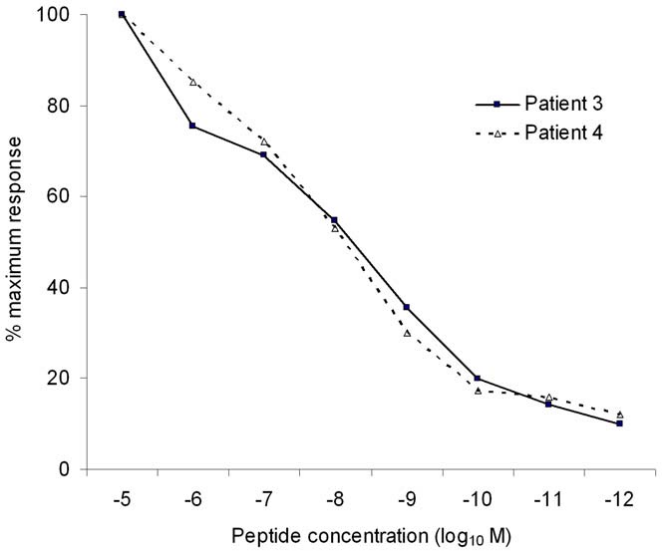
Dose-response titration of the peptide CAPAGFAIL. The responsiveness of T cells from two HIV-infected individuals to 10-fold dilutions of the peptide CAPAGFAIL was assessed by IFN-γ ELISPOT assay. The results shown are the specific response elicited at each peptide concentration, expressed as a percentage of the maximum response (that elicited by 10^−5^ M peptide) observed in the individual concerned.

We then tested whether any of these HLA-Cw*0102-binding peptides were recognised by virus-specific T cells from HIV-1-infected individuals. Peripheral Blood Mononuclear cells (PBMCs) from 5 HLA-Cw*0102-positive individuals chronically-infected with clade B viruses were screened for reactivity to the 11 peptides using IFNγ ELISPOT T cell assays. To conserve patient PBMCs, the 11 peptides were initially split into 3 pools (Pool 1: peptides 1–4; Pool 2: 5–8; Pool 3: 9–11). Two of the five subjects (patients 3 and 4) responded to Pool 1 (Figure 2a); and subsequent testing of T cell reactivity to the 4 individual peptides within this pool confirmed peptide 1 as the epitope sequence recognised by both of these patients (Figure 2b). Peptide 1 was the HIV peptide with the highest affinity of binding to HLA-Cw*0102 (CAPAGFAIL; pBL_50_ = 8.0). In order to assess the avidity of the patient T cell responses to this epitope, we also titrated the epitope peptide in IFN-γ ELISPOT assays using PBMCs from the responding patients. In both individuals, the response to CAPAGFAIL had an avidity of approximately 10^−8^ M (Figure 3). Using our QSAR-based model for prediction of peptide binding to HLA-Cw*0102, we were thus able to identify a HLA-Cw*0102-restricted T cell epitope in the human pathogen HIV-1 to which high-avidity responses could be detected in a subset of individuals chronically-infected with this virus.

## Discussion

In this paper we have used a potent combination of *in silico* prediction and *in vitro* verification to characterise the peptide binding specificity of the previously poorly- characterised human MHC molecule HLA-Cw*0102. A 2D-QSAR approach was used to model binding of nonameric peptides to HLA-Cw*0102 [**19**]. We defined positive and negative contributions made to HLA-Cw*0102 binding affinity by amino acids at all positions of the nonameric peptide sequence. This model was employed to predict four “optimised” HLA-Cw*0102-binding peptides, all of which showed a high affinity of binding to HLA-Cw*0102. Alanine-scanning was used to probe and weight the contributions to affinity of amino acids at each position of a HLA-Cw*0102-binding peptide. Furthermore, the importance of amino acids randomly allocated at each position of the peptide was explored by evaluating the experimentally-determined binding affinities of a set of sequence-distinct peptides defined by discrimination of binding and diversity analysis. Realising that prediction must be verified both *in vitro* and *ex vivo*, we also validated the utility of our approach by predicting HLA-Cw*0102-binding nonameric peptides in the HIV-1 sequence, one of which was confirmed to be an epitope recognised by virus-specific T cells in a subset of HIV-infected HLA-Cw*0102-positive individuals.

MHC class I molecules demonstrate extensive polymorphism across the human population, even at the HLA-C locus. Four mechanisms are largely responsible for the creation of new MHC alleles: point substitution, allele conversion, gene conversion, and recombination. The size and diversity of the MHC repertoire is problematic for immunoinformatics. One solution is to identify supertypes [**34]**, [42****], another is the development of methods that directly infer the selectivities of uncharacterised alleles [**33]**, [43], [44****], and another is the *de novo* use of molecular dynamics [**45]**, [46****]. Here, we used a combination of computational and experimental approaches to develop a hybrid *in silico-in vitro* algorithm for predicting peptide binding to the MHC class I molecule HLA-Cw*0102. The analysis of poorly-characterised alleles, such as HLA-Cw*0102, is important in this context as it allows such approaches to be validated and calibrated. Moreover, the data-driven methods devised here can yield important results in their own right.

The binding specificity of HLA-Cw*0102 has previously been characterised by sequence motifs [**28]**, [29], [30****]. However, there are many problems with the use of such motifs. The most significant of these is that motifs are, fundamentally, deterministic. A peptide is either a binder or is not a binder. Generally speaking, the presence of anchors, frequently located at positions 2 and/or 9 of a nonameric peptide, is deemed to be necessary, but not sufficient, for high affinity binding [**6**]. Prominent roles for other positions, typically 1, 3, and 7 (so-called secondary anchor residues) are also commonly observed [**47**]. It is well-known that matches to motifs produce many false positive peptides that fail to bind to MHC, and are, in all probability, producing an equal number of false negatives, although peptides predicted to be non-binders are seldom screened. Hence, although motif-based approaches to prediction of peptide MHC binding specificity have proven useful, they are increasingly seen as inadequate. An alternative approach that we have pursued in previous studies is to employ methods that dissect the contributions of amino acids at all different positions of the peptide (which tend to weight the positions equally) in order to predict peptide-MHC interactions [**6]**, [7], [9****]. Likewise here, we again used a QSAR-based approach to quantify the contribution to HLA-Cw*0102 binding affinity made, on a position-wise basis, by amino acids along the whole of a nonameric peptide. We found that amino acids at all positions in the peptide could exert a positive or negative influence on peptide binding to MHC, a finding that highlights the complexity of peptide-MHC interactions. This was further emphasised by our observation that a series of alanine-scanning mutants of the high-affinity HLA-Cw*0102-binding peptide NCPERIITL all bound to HLA-Cw*0102 with affinities equivalent to or higher than those of the index peptide, again suggesting that peptide binding to HLA-Cw*0102 is not predominantly dependent on any single residue, but rather is determined by multiple interactions between the peptide and MHC molecule. These findings underline the shortcomings of approaches based on simple motifs for prediction of peptide binding affinities, and provide a rationale for the use of more sophisticated and insightful alternative approaches such as the QSAR-based method employed in this study.

Cross-validation of our 2D-QSAR-based model for peptide binding to HLA-Cw*0102 indicated that it provided good predictions of the HLA-Cw*0102-binding affinities of the peptides in Table 1. As seen previously in our analysis of HLA-A*0201 [**10**], there was a trend for the experimentally-determined peptide binding affinities to be somewhat higher than their predicted binding affinities. Synergies operate between different peptide positions, and this may help to explain the underestimated affinities seen here and previously [**10]**, [48****]. The observation that many of the peptides had higher binding affinities than suggested by a summation of amino acid contributions is an example of co-operative enthalpy-entropy compensation [**49**]. It is now well known that the balancing of intermolecular motion and enthalpic interactions induces a non-linear amplification of binding affinity. Intermolecular motion weakens the many non-covalent interactions that stabilize a complex. As additional interactions are introduced, intermolecular motion is damped. As a result, all interactions are more favorable and the complex is more cohesive. As an example, peptide 17 (Table 1) is a medium binder, while peptide 35 is a non-binder, yet the sequence is the same except for the substitution of an arginine in peptide 35 by a proline in peptide 17 at peptide position P4. Examination of Table 2 and Figure 1 indicates that proline at P4 results in an overall unfavourable contribution to binding, while arginine makes a contribution which is overall nearly neutral. On this basis, peptide 17 would be expected to be a less good binder than peptide 17, whilst the converse is in fact the case. This apparent albeit minor inconsistency may be due to a cooperative or enthalpy-entropy compensation effect.

Unfortunately, intrinsic error in the biological measurements precludes construction of a predictive method that can effectively factor-out these co-operative interactions. Instead, the signal is suppressed within a low signal-to-noise ratio: use of a model with more variables does not yield better statistics. There are a number of factors that may limit the accuracy of the method used for analysis of peptide-MHC binding affinity and prevent use of the data to probe such subtle effects with confidence. In this context, it is worth noting that the recognition of class I MHC molecules by HLA allele-specific antibodies can be influenced by the identity of bound peptides. It is thus possible that the Cw*0102-specific mAb VP6G3 used in this study may bind to distinct peptide-MHC complexes with different affinities in a peptide-dependent manner. It was beyond the scope of the present study to evaluate such binding in a fully quantitative manner. On the basis of other studies such discrepancies are unlikely to be significant; yet we can not dismiss this phenomenon, which may contribute to the noise inherent within the biological observations and thus the imprecision of any derived prediction method.

The QSAR-based model was used to predict four “optimised” HLA-Cw*0102-binding peptides. All of these showed a high affinity of binding to HLA-Cw*0102, although their binding affinities did not exceed those of the most affine peptide in the original peptide set. This may have been because the contributions of all amino acid types at all positions were not adequately represented in the dataset used to generate the model. This is a shortcoming common to traditional natural-peptide-only analyses [**6**], which leads to highly biased and restricted distributions of amino acids. Here, we explored the role of different amino acids at positions throughout the peptide by generating a set of sequence-diverse peptides and measuring their binding to the HLA-Cw*0102 molecule. The overall model comprising all affine peptides did not improve on the initial model. There are many possible reasons for this. The set of peptides may, as a consequence of its diversity, actually comprise several subsets with distinct structure-activity relationships. Measured binding may contain some element of cross- or non-specific binding to other cell surface molecules. Nonetheless, the high proportion of binders observed provides significant validation for the efficiency of this approach. In future, discriminant models - as well as affinity models – could generate diverse peptide sets iteratively leading to more comprehensive and robust QSAR-based models for peptide binding to HLA-Cw*0102 or, more generally, for any allele.

To validate the utility of our QSAR-based model for prediction of HLA-Cw*0102-binding peptides in a pathogen sequence that may constitute epitopes recognised by the host immune response, we tested the ability of the model to predict putative nonameric HLA-Cw*0102-restricted T cell epitopes in the HIV-1 sequence. There were several reasons for choosing HIV-1 as a test pathogen. First, there is a strong rationale for identifying HIV-1 peptides presented by distinct MHC class I molecules that may be recognised by T cells and/or NK cells *in vivo*. Identification of regions of the HIV-1 genome that are targeted by the immune system is a valuable asset for basic research aimed at characterising the host immune response plus viral immune evasion strategies and identifying correlates of immune protection, knowledge that is urgently needed to inform the development of new prophylactic and therapeutic vaccination strategies to combat this infection [**50]**, [51****]. Second, HIV sequence data is readily available [**41**], further, as a virus with a reasonably small (∼9.2 kb) genome, it was feasible to synthesise and test all the predicted HLA-Cw*0102-binding peptides in the proteome. Given the extent of HIV-1 sequence diversity, we confined our analysis to work on clade B viruses/virus-infected individuals, and predicted HLA-Cw*0102-binding peptides in both the clade B concensus virus sequence and the sequence of a “reference” clade B virus, HXB2. Finally, HIV-specific T cell frequencies in healthy, viremic individuals are typically high enough for responses to epitopes of varying immunodominance to be detected *ex vivo* by standard immunological assays, facilitating screening of HLA-Cw*0102-binding peptides for T cell recognition.

Eleven HLA-Cw*0102-binding nonameric peptides were identified in the HIV-1 clade B concensus and/or HXB2 sequences, one of which, CAPAGFAIL, was shown to be recognised by HIV-specific T cell responses of high avidity in two of the five HLA-Cw*0102-positive individuals chronically infected with HIV that we tested. Subsequent to the completion of this study, the same nonameric sequence was also described as a HLA-Cw*0102-restricted T cell epitope recognised in other HIV-infected individuals [**52**]. It is notable that two of the eleven HLA-Cw*0102-binding nonameric peptides we identified (peptides 4 and 5 in Table 4) contained an octameric sequence, VIPMFSAL, that has previously been shown to constitute an epitope recognised by the T cell response in HIV-infected individuals [**23]**, [31****]. The lack of recognition of peptides 4 and 5 by T cells from the HIV-infected subjects we studied may reflect a lack of reactivity to this epitope-containing region in these individuals or an inability of VIPMFSAL-specific T cells to recognise complexes of the longer peptides bound to HLA-Cw*0102. However, it is most likely to result from the poor presentation of the VIPMFSAL-containing nonameric peptides in our assays. Both peptides had moderate to low HLA-Cw*0102 binding affinities and may have been out-competed by endogenous peptides for presentation on PBMCs. Studies in which HIV-specific T cell responses were mapped empirically [**23]**, [31****] have identified HLA-Cw*0102-restricted T cell responses to additional HIV-1 clade B peptides that were not identified as putative HLA-Cw*0102-restricted HIV T cell epitopes in this study. Potential explanations are many. First, the optimal sequences of some of these epitopes are not nonamers (and our model predicts only nonameric HLA-Cw*0102-binding peptides). Second, as discussed above, all models, including ours, provide only moderately successful predictions of all potential HLA-Cw*0102-binding peptides, hence the binding affinity of certain peptides may have underestimated. Finally, a subset of epitopes recognised by virus-specific T cells may bind to MHC with very low affinity, and hence may not have been characterised as HLA-Cw*0102-binding peptides by our model.

Had we been able to test more HLA-Cw*0102-positive patients, we might potentially have identified T cell responses to more of the HLA-Cw*0102-binding peptides in Table 4. The epitopes targeted by the HIV-specific T cell response in a given infected individual are determined by host factors - antigen processing, the complement of HLA class I molecules expressed, and the profile of T cell receptors in the repertoire (which will have been modified by the infection and vaccination history of the subject, as well as by their individual genetics) - and also by the sequence of the *in vivo* viral quasispecies, which not only varies markedly from one infected individual to another, but also undergoes considerable evolution within a given subject over time [**52**]. In particular, detection of responses to those HLA-Cw*0102-binding peptides in poorly conserved regions of the viral proteome may require screening of a larger number of subjects, as the test peptide sequences may not be well-conserved in the viral quasispecies present in all individuals. It was notable that the CAPAGFAIL peptide to which responses were observed in two subjects was the HIV peptide that exhibited the highest affinity of binding to HLA-Cw*0102 (Table 4). Responses to other lower-affinity peptides may have been rarer and/or less immunodominant, requiring screening of a large patient cohort for their detection. The T cell responses we detected to the CAPAGFAIL epitope were also of high avidity, so may have been better-able to cross-recognise our test peptide in spite of sequence differences to the autologous virus sequence than lower avidity responses to other viral epitopes. The patient samples used to screen for T cell responses to our HLA-Cw*0102-binding peptides were derived from individuals chronically-infected with HIV, as the breadth of epitope recognition in chronic infection is greater than that in acute infection [**54**]. However given the increasing evidence to suggest that there are differences in the epitopes to which strong responses are detectable in the acute and chronic phases of infection, with a subset of the initially-immunodominant responses undergoing a rapid decline in frequency, due in part to viral mutational escape and loss of the epitope sequence [**55]**, [56****], it would also have been of interest to determine whether responses to additional HLA-Cw*0102-binding peptides were detectable in acutely-infected individuals: unfortunately, suitable samples were not available.

Another question of interest is whether any of the HLA-Cw*0102-binding HIV-1 peptides we identified may form part of a peptide-MHC complex recognised by NK cell receptors in HIV-infected individuals. HLA-Cw*0102 is a HLA-C1 serogroup allele that interacts with the inhibitory KIR2DL2/3 and activating KIR2DS2 receptors [**57**]. In addition, HLA-C molecules act as ligands for other NK receptors including ILT2 and CD160 [**58**]. Current thinking suggests that the peptide content of MHC class I molecules may modify their interaction with activating and inhibitory NK cell receptors, such that predominantly inhibitory signals are delivered when NK cells interact with MHC molecules presenting self peptides on normal body cells, but a more activating balance of signals are delivered when NK cells interact with MHC molecules presenting viral or stress peptides on infected/transformed cells [**59**]. A recent study showed that a HLA-Cw4-restricted HIV-1 CD8+ T cell epitope could also be recognised by the NK cell receptor KIR2DL1 [**60**]; so by analogy, it is possible that some of the HLA-Cw*0102-binding peptides we identified in this study may constitute KIR2DL2/3 or KIR2DS2 ligands.

In conclusion, we have used a combination of computational and *in vitro* techniques to illuminate the characteristics of peptides presented by HLA-Cw*0102. We show how the analysis of a relatively small number of peptides can power a predictive algorithm capable of scanning a viral genome and identifying binding peptides from which an epitope emerges. What we really require are reliable high-throughput screening approaches able to generate large-scale data sets, such as that recently reported by Buus and co-workers [**61**]. However, and despite such caveats, we have shown clearly that even with a relatively modest low-throughput approach one can generate models with proven utilitarian value.

## Materials and Methods

### Patients and Blood Samples

Individuals chronically infected (>6 months) with HIV-1 (clade B) were recruited from the Centre for Sexual Health and HIV Research (London, UK). Ethical approval for these studies was obtained from the National Health Service Camden and Islington Community Local Research Ethics Committee, and blood samples were drawn with written informed consent. Patients were asymptomatic and typically not receiving anti-retroviral therapy at the time of sample acquisition. Blood was collected into acid citrate dextrose or EDTA. PBMCs were separated by centrifugation over a Histopaque 1.077 density gradient (Sigma-Aldrich, Poole, UK) and cryopreserved until needed.

### HLA Class I Typing

DNA was isolated from patient PBMCs using a QIAamp DNA Blood Mini Kit (Qiagen Ltd, Crawley, UK). HLA class I fine typing was largely performed at the Churchill Hospital (Oxford, UK) using a molecular PCR method employing sequence-specific primer mixes.

### Peptides

43 nonameric peptide sequences used in the study were abstracted from Barber *et al*. [**28**] and Andersen *et al.* [**23**] (Table 1). We tested only nonameric peptides, so overlapping peptides were obtained where only longer peptides were reported. All peptides used in this study were ordered either from Mimotopes (Pensby, UK) or from the in-house peptide synthesis service at the Institute for Animal Health (Compton, UK).

### Peptide Binding Assay

Peptide binding to HLA-Cw*0102 was assessed using a FACS-based MHC stabilization assay [**62**] with modifications as described elsewhere [**36**]. This assay involves use of the transporter associated with antigen processing (TAP)-deficient cell line T2, which has very low levels of surface MHC class I expression due to the poor stability of non-peptide-loaded MHC class I molecules. Incubation of T2 cells in the presence of peptides able to bind to one or more of the MHC class I molecules expressed by these cells (HLA-A*0201, HLA-B5 and HLA-Cw*0102) stabilizes their expression and results in an increase in surface MHC class I levels that can be measured by flow cytometry. In this study, a HLA-Cw*0102-specific antibody was used to measure peptide-induced stabilization of HLA-C expression on T2 cells, hence allowing evaluation of peptide binding to HLA-Cw*0102. Briefly, T2 cells were incubated in 96-well flat-bottom plates at 2x10^5^ cells per well in a 200 µl volume of AIM V medium (Life Technologies, Paisley, UK) with human β_2_-microglobulin at a final concentration of 100 nM (Scipac, Sittingbourne, UK) with and without peptides at concentrations between 200 and 0.04 µM for 16 h at 37°C. Cells were then washed and surface levels of HLA-Cw*0102 were assessed by staining with Cw*0102-specific mAb VP6G3 [**63**] and a FITC-conjugated AffiniPure F(ab')_2_ fragment goat anti-human IgM Ab (Jackson Immunoresearch Laboratories, West Grove, PA, USA). Cells were fixed at 4°C in 4% paraformaldehyde and analyzed on a FACSCalibur (BD Biosciences, Oxford, UK) using CellQuest software. Results are expressed as fluorescence index (FI) values. These were calculated as the test mean fluorescence intensity (MFI) minus the no peptide isotype control MFI divided by the no peptide HLA-Cw*0102-stained control MFI minus the no-peptide isotype control MFI. The half-maximal binding level (BL_50_), which is the peptide concentration yielding the half-maximal fluorescence intensity (FI) of the reference peptide in each assay, was calculated and is presented as pBL_50_ (–logBL_50_). The peptide VIPMFSAL, which binds with high affinity to HLA-Cw*0102, was used as a reference peptide [**23**]. Peptides with a pBL_50_ above 6.0 were designated as high binders, between 4.5 and 6.0 as medium binders, between 3.0 and 4.5 as low binders, and below 3.0 as non-binders.

### Additive 2D-QSAR Method

Two models using the additive method were developed: the first contained only the amino acid contributions (eqn. 1) and the second contained both amino acid contributions and side chain – side chain interactions (eqn. 2): 
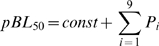
(1)

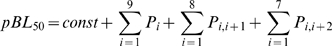
(2)where the *const* accounts for the peptide backbone contribution, 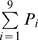 is the sum of amino acid contributions at each position, 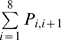 the sum of adjacent peptide side-chain interactions, and 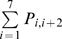 the sum of interactions between every second side-chain.

Every peptide sequence was presented as a string of 0s and 1s with a length of 180 terms (20 aa×9 positions) or 6180 terms (20×9+20×20×8+20×20×7). A term is equal to 1 when a certain amino acid or interaction at a certain position exists, and 0 when it is absent. Columns containing only 0s were omitted. The pBL_50_ values (dependent variables) were included in the matrix as a first column.

The partial least squares (PLS) method was used to solve this matrix. PLS handles data matrices with more variables than observations by forming new *x* variables, named principal components, as linear combinations of the old ones and then uses them as predictors of the biological activity. We used the PLS method as implemented in the QSAR module of SYBYL6.9 [**64**]. pBL_50_ was put as a dependent variable. The scaling method was set to “none”. The column filtering was switched off. The optimal number of components was found by leave-one-out cross-validation. The predictive power of the model was assessed by the cross-validated coefficient *q^2^* and standard error of prediction: 
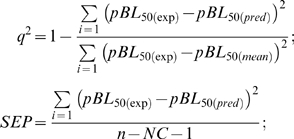
where pBL_50(pred)_ is the predicted pBL_50_ of the excluded peptide and n the number of peptides.

The non-cross-validated model was assessed by the explained variance *r^2^* and standard error of estimate: 
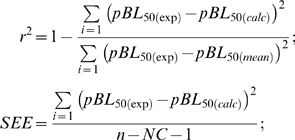
where pBL_50(calc)_ is the calculated pBL_50_ of the non-excluded peptide. The non-cross-validated model was used to predict the binding affinity of newly designed peptides.

### IFNγ ELISPOT Assay

Multi-Screen plates (MAHAS4510, Millipore Ltd, Watford, UK) were coated overnight at 4°C with 5 µg/ml anti-human IFNγ capture antibody 1-D1-K (MabTech AB, Nacka, Sweden) in PBS. Plates were washed 3 times with sterile PBS and blocked with 10% heat-inactivated pooled human serum (PHS) (Sigma, UK) in RPMI (sterile filtered) for 1 hr at 37°C in 5% CO_2_. Patient PBMCs were thawed and washed 3 times in RPMI supplemented with 15% FCS (Invitrogen Life Technologies, Paisley, UK), and then resuspended in RPMI containing 5% PHS and dispensed into plates at 2×10^5^ cells/well in a 100 µl volume. Cells were stimulated in duplicate with medium only, PHA (Sigma-Aldrich, Gillingham, UK) at 10 µg/ml or specific peptide at 10^−5^ µM in a final volume of 120 µl for 1 hour at 37°C in 5% CO_2_. 30 µl of filtered FCS was then added to each well and the plates were incubated at 37°C in 5% CO_2_ for 16–20 hours. Plates were washed 3 times with PBS/0.05%Tween-20 and incubated for 2 hours at 37°C in 5% CO_2_ with 1 µg/ml biotinylated anti-human IFNγ detection antibody 7-B6-1 (MabTech AB) in PBS. Plates were then washed 3 times with PBS/Tween-20 prior to a 1-2 hour incubation at room temperature with a 1/1000 dilution of alkaline phosphatase anti-biotin (Vector Laboratories, Peterborough, UK) in PBS/Tween-20. Following washing, enzyme activity was detected using an alkaline phosphatase conjugate substrate kit (Bio-Rad, Hemel Hempstead, UK) following the manufacturer's protocol and plates were incubated at room temperature for 30 minutes. The reaction was stopped by rinsing the plates 3 times in tap water. Plates were air-dried and spots enumerated using an AID image analysis system with AID ELISPOT software version 2.5 (Autoimmune Diagnostika GmbH, Strassberg, Germany). A positive response was defined as one where the average number of spot forming cells was at least twice the background number of SFC in medium alone wells and exceeded 20 spots per 10^6^ PBMC.
